# Documenting clinical performance problems among medical students: feedback for learner remediation and curriculum enhancement

**DOI:** 10.3402/meo.v18i0.20598

**Published:** 2013-07-22

**Authors:** Brian E. Mavis, Dianne P. Wagner, Rebecca C. Henry, Laura Carravallah, Jon Gold, Joel Maurer, Asad Mohmand, Janet Osuch, Steven Roskos, Andrew Saxe, Aron Sousa, Vince Winkler Prins

**Affiliations:** 1 Office of Medical Education Research and Development, Michigan State University College of Human Medicine, East Lansing, MI, USA; 2 Department of Medicine, Michigan State University College of Human Medicine, East Lansing, MI, USA; 3 Department of Pediatrics and Human Development, Michigan State University College of Human Medicine, Hurley Medical Center, Flint, MI, USA; 4 Department of Pediatrics and Human Development, Michigan State University College of Human Medicine, East Lansing, MI, USA; 5 Department of Obstetrics Gynecology and Reproductive Biology, Michigan State University College of Human Medicine, East Lansing, MI, USA; 6 Department of Medicine, Virginia Commonwealth University Health System, Richmond, VA, USA; 7 Departments of Surgery and Epidemiology, Michigan State University College of Human Medicine, East Lansing, MI, USA; 8 Department of Family Medicine, Michigan State University College of Human Medicine, East Lansing, MI, USA; 9 Department of Surgery, Michigan State University College of Human Medicine, East Lansing, MI, USA; 10 Department of Family Medicine, Georgetown University, Washington, DC, USA

**Keywords:** OSCE, performance problems, skills remediation, faculty ratings, checklist vs global ratings

## Abstract

**Introduction:**

We operationalized the taxonomy developed by Hauer and colleagues describing common clinical performance problems. Faculty raters pilot tested the resulting worksheet by observing recordings of problematic simulated clinical encounters involving third-year medical students. This approach provided a framework for structured feedback to guide learner improvement and curricular enhancement.

**Methods:**

Eighty-two problematic clinical encounters from M3 students who failed their clinical competency examination were independently rated by paired clinical faculty members to identify common problems related to the medical interview, physical examination, and professionalism.

**Results:**

Eleven out of 26 target performance problems were present in 25% or more encounters. Overall, 37% had unsatisfactory medical interviews, with ‘inadequate history to rule out other diagnoses’ most prevalent (60%). Seventy percent failed because of physical examination deficiencies, with missing elements (69%) and inadequate data gathering (69%) most common. One-third of the students did not introduce themselves to their patients. Among students failing based on standardized patient (SP) ratings, 93% also failed to demonstrate competency based on the faculty ratings.

**Conclusions:**

Our review form allowed clinical faculty to validate pass/fail decisions based on standardized patient ratings. Detailed information about performance problems contributes to learner feedback and curricular enhancement to guide remediation planning and faculty development.

## Background

With increased concern about patient safety and medical error (1), educators are focusing not only on what learners know but also on what learners do (2), and as a result medical education has embraced performance-based assessment. As discourse about the social contract between medical education and the public gains broad acceptance, accountability and transparency have become the language of accreditation and licensure. Perhaps, this is best summed up by Shumway and Harden (3):

Society has the right to know that physicians who graduate from medical school … are competent and can practice their profession in a compassionate and skillful manner. It is the responsibility of the medical school to demonstrate that such competence has been achieved … Assessment is of fundamental importance because it is central to public accountability. (p. 569)

A survey by Hauer and colleagues in 2005 reported that 84% of medical schools had implemented a comprehensive clinical skills assessment with 70% requiring pass in the examination for a graduation. They also observed that 67% of faculty respondents believed that the implementation of a national clinical skills examination increased the importance for medical schools to conduct their own clinical skills assessments (4).

The United States Medical Licensing Examination (USMLE) Step 2 CS (clinical skills) examination sets an important external achievement bar for graduation, but by its nature, provides students and institutions with little useful feedback for diagnosing performance gaps and remediating student performance. Furthermore, there is insufficient detail in the Step 2 CS institutional reports to identify related curricular insufficiencies. For that guidance, medical schools must create their own assessment methods to diagnose and remediate gaps in students’ clinical performance.

A more recent study by Hauer and colleagues (5) reported that while comprehensive clinical skills examinations have been adopted by many medical schools, relatively little is known about the types of performance problems demonstrated by students during these experiences. Clearly, checklists used in simulated encounters provide curricular feedback about the adequacy of specific performance elements of communication, history taking, physical examination and procedural skills. These ratings typically are interpreted as representing the extent to which the learner has demonstrated competent performance for the specific tasks required in each encounter. Students who fail to demonstrate competency seldom get much corrective feedback since the specific checklist items often are not revealed in order to preserve the security of the clinical case for future examinees. Furthermore, skills checklists are limited in that skills are disaggregated into discrete steps representative of how a novice might approach a task (6, 7). Checklists inherently limit the range of judgments available to raters, who can provide feedback only on the specific elements of the task, but not on the quality or organization of these elements (6). While ratings by physician and non-physician raters yield comparable scores when checklist-based scores are compared, there is evidence that global clinical performance ratings are more accurate when completed by trained physician examiners than non-physician raters (8). Raters with less experience pay more attention to specific elements of performance compared to expert raters, who attend to contextual factors and consider performance more broadly (9).

After interviewing a sample of faculty experienced with comprehensive clinical skills assessments, Hauer et al. (5) proposed a taxonomy of performance problems commonly demonstrated by students participating in these events. Furthermore, their survey revealed that many of the problems encountered by students would not be captured by simple task checklists. They characterized three broad types of performance problems: technique problems related to specific task performance (e.g., examined patient through the gown), cognitive problems associated with gathering data (e.g., premature closure of diagnostic options), and non-cognitive problems often manifested as issues of professionalism (e.g., treated patient as a diagnostic problem rather than a person with feelings and concerns). To the extent that these kinds of performance problems can be consistently identified, they can be used for diagnostic purposes to inform clinical skills instruction and assessment as well as remediation efforts required for poorly performing students (5).

This study extends the 2007 work of Hauer and colleagues (5), which characterized the types of performance problems common to students in clinical competency examinations. This study refines their taxonomy using a specially created worksheet that characterizes common performance problems observed among medical students. The worksheet provides more useful information than the checklists commonly used by standardized patients in simulation settings, and serves as a guide for faculty involved in providing formative feedback or planning remediation for students who fail to demonstrate competency on summative assessments. This worksheet has the added advantage of not being case-specific like the standardized patient (SP) checklists, and preserves the security of high-stakes clinical competency examinations. For programmatic evaluation, it provides a structured approach for obtaining quantitative information about the frequency of common medical student performance problems.

Also of interest in this study was the extent to which faculty ratings could be used to reproduce the final examination results derived from SP ratings. Could faculty ratings and SP ratings support the same pass/fail decisions of learner performance? Ratings from SPs are routinely used in objective structured clinical examinations (OSCEs) as the basis for pass/fail decisions. While SPs are typically well-trained for the task, they are not clinicians, and student outcomes based on SP ratings could be validated if ratings from expert faculty educators supported the pass/fail decisions.

Specifically, the purpose of this article is to: (1) describe the development and pilot testing of a faculty worksheet characterizing common medical student performance problems; (2) determine the frequency of specific performance problems evident among third-year medical students; (3) discover the extent to which faculty ratings reproduce the pattern of passes and failures originally based on SP ratings; and (4) examine the extent to which identified performance problems are found to vary across different clinical case scenarios.

## Methods

### Context

Since 2006, all Michigan State University College of Human Medicine students have been required to participate in a comprehensive clinical skills OSCE examination at the end of their third-year required clerkships. Since 2008, the examination has been summative, requiring students to demonstrate skills at a minimum standard. The OSCE had eight stations: six stations were graded encounters contributing to pass/fail decisions and two stations that pilot tested new cases. Each graded station involved a 20 min encounter with an SP and 10 min to complete a post-encounter note. The ratings for each station were completed by the standardized patient, using a structured checklist of communication, history-taking, and physical examination skills specific to the encounter. To move from a low-stakes to a summative examination and based on prior standard-setting exercises, our faculty had determined that to pass each case a student was required to demonstrate minimum competence independently in the communication, history-taking, and physician examination components. To pass the OSCE examination overall, the student was required to meet the competency standards for at least four out of six graded cases.

### Clinical encounters

The clinical encounters used in this study were selected from the results of all third-year medical students (*N*=97) who had participated in the comprehensive clinical skills examination described above during the final year in which it was a low-stakes examination. Based on analysis of the SP ratings and using the grading criteria described above, there were 41 (42%) students who did not demonstrate minimum competency for at least four of the six graded cases. These 41 students together failed to achieve minimal competency scores in a total of 132 clinical encounters; these encounters were assigned to faculty pairs for ratings.

### Rating worksheet

A performance problem worksheet was developed from the qualitative study published by Hauer and colleagues (4) reporting the results of interviews with faculty involved in local clinical skills assessments. Based on their published account of technical, cognitive and non-cognitive problems, a draft list of technical and cognitive performance problems related to the medical interview and the physical examination was developed. A number of the performance problems identified (4) were not amenable for use in an observational rating task, for example, ‘generated correct differential diagnosis’. The draft items were independently reviewed by a second faculty member to assure that the performance problem items were both observable and amenable to rating from observation of digital video recordings. The final version of the rating worksheet listed 26 items organized into three sections: medical interview (14 items), physical examination (7 items), and professionalism (5 items), as shown in the Appendix. A final overall assessment of student performance at the end of the worksheet was used to record the rater's final decision about each student's performance. Faculty rated each item on a three-point scale: unsatisfactory, marginal, or satisfactory. A prior faculty standard-setting exercise determined that ratings of ‘marginal’ had sufficient concerns to be categorized as ‘unsatisfactory’. Therefore, all subsequent analyses were based on dichotomous item ratings as either unsatisfactory or satisfactory.

### Raters and rater training

A core group of 10 experienced faculty clinician-educators were recruited as raters for this study. They represented faculty in various roles who were responsible for curriculum oversight and implementation, such as clerkship directors, clinical skills teaching faculty, and clinical educators involved with the preclinical curriculum. Orientation and training of faculty raters was conducted over three two-hour sessions during which six clinical encounters from the prior year's OSCE examination were viewed as a group. The six encounters represented two students each whose case performance based on SP ratings were below average, average, and above average. For this exercise, faculty raters were blinded to the results of the SP ratings. Ratings were completed independently and were tallied. Faculty members then discussed their ratings as well as any specific issues or concerns. This iterative process was used for all six pilot test cases.

The final rating task for this project required that each clinical encounter be independently rated as satisfactory, marginal, or unsatisfactory by two faculty members, consistent with an expert judgment approach. The paired ratings of each encounter represented the independent expert judgment of two faculty raters.

This study was determined to be exempt by the university institutional review committee.

## Results

### Pilot test rater agreement of performance problems

The aggregate ratings of satisfactory/unsatisfactory averaged across the six pilot test cases were used as an index of rater agreement for each item. As previously stated, ratings of marginal were considered as unsatisfactory. The rating items and aggregate rater agreement are shown in Table 1. Agreement ranged from 63 to 98% with an average of 78%; none of the ratings reached 100% agreement. The items in the Professionalism section had the highest agreement scores. Of the 26 total items on the worksheet, all but three had agreement scores of 70% or greater. Two of the items that did not reach 70% agreement were associated with technical aspects of the medical interview: use of open-ended questions, and use of clarifying or follow-up questions. A third item – inadequate data-gathering – was used to describe the physical examination. The overall assessment of passing/failing the clinical encounter had 70% agreement.

**Table 1:** Pilot test faculty rater agreement

1A. Medical interview: technique problems		Rater agreement (*N*=10 faculty) (%)
1	Required elements are missing	82
2	Approach lacks structure or order	77
3	Approach lacks flow or transitions	76
4	Did not use clarifying questions or follow-up on positive responses	63
5	Little or no use of open-ended questions	63
6	Rushed through data gathering; did not use available time	83
		
1B. Medical interview: cognitive problems		
1	Inadequate history to rule out other possible diagnosis	72
2	Premature closure of diagnostic options	71
3	Inadequate characterization of social history	78
4	Inadequate characterization of family history	83
5	Inadequate characterization of problem/symptom dimensions	77
6	Inadequate characterization of past medical history	73
7	Inadequate characterization of chief complaint	73
8	Medical interview covered areas not indicated by the case	90
		
2A. Physical examination: technique problems		Rater agreement (*N*=10 faculty)
1	Required elements are missing	78
2	For physical examination elements performed technique was poor	73
3	Examined patient over gown	90
4	Approach lacks structure or order	72
5	Rushed through examination; did not use available time	76
		
2B. Physical examination: cognitive problems		
1	Inadequate data gathering to rule out other possible diagnosis	64
2	Employed examination maneuvers not indicated by the case	75
		
3. Professionalism		Rater agreement (*N*=10 faculty)
1	Student did not introduce self to patient	93
2	Treated SP as diagnostic problem rather than person with feelings and concerns	80
3	Student did not wash hands	85
4	Poor verbal and non-verbal communication (emotional distance)	84
5	Student dressed inappropriately or casually	98
		
4. Overall assessment: failure of clinical encounter		70

### Defining performance problems

The 132 failed clinical encounters were assigned to faculty pairs for ratings; a total of 82 encounters (62% of all failed encounters) had two independent ratings by faculty members. Not all faculty raters completed their rating task: 42 encounters were rated by only one faculty member and eight encounters were unrated. The remaining analyses are based on the 164 paired ratings of 82 encounters.

The final decision of whether a student passed or failed the examination was based on the final overall assessment of student performance. There was an 85% agreement between faculty pairs on the final overall assessment of student performance.

### Prevalence of performance problems

Table 2 summarizes the frequency of performance problems among this cohort of students across 82 clinical encounters. Eleven out of the 26 performance problems listed on this worksheet were present in 25% or more of the observed encounters. For the medical interview, the most prevalent problem identified was an inadequate history to rule out other possible diagnoses, with more than half of the students (60%) identified in this category. Of the top five problems identified in the medical interview, only one was associated with technique, with the remainder associated with insufficient data collection. Overall, 37% of the encounters had an unsatisfactory medical interview with one or more performance problems identified.

**Table 2:** Prevalence of performance problems among M3 students

			Prevalence by case			
			
		Problem prevalence (%)	Case 1 (%)	Case 2 (%)	Case 3 (%)	Probability
1A. Medical interview: technique problems						
1	Required elements are missing	25	18	44	0	.007
2	Approach lacks structure or order	16	0	29	5	.029
3	Approach lacks flow or transition	15	0	29	0	.006
4	Did not use clarifying questions or follow-up on positive responses	10	0	5	17	NS
5	Little or no use of open-ended questions	7	0	18	5	NS
6	Rushed through data gathering; did not use available time	2	0	12	0	NS
						
1B. Medical interview: cognitive problems						
1	Inadequate history to rule out other possible diagnosis	60	85	73	33	NS
2	Premature closure of diagnostic options	41	79	43	12	.001
3	Inadequate characterization of social history	38	21	74	6	.001
4	Inadequate characterization of family history	35	25	57	5	.002
5	Inadequate characterization of problem/symptom dimensions	23	8	29	22	NS
6	Inadequate characterization of past medical history	17	8	26	11	NS
7	Inadequate characterization of chief complaint	6	0	22	0	.022
8	Medical interview covered areas not indicated by the case	1	8	0	0	NS
			Prevalence by case			
			
		Problem prevalence	Case 1	Case 2	Case 3	Probability

2A. Physical examination: technique problems						
1	Required elements are missing	69	91	57	82	NS
2	For physical examination elements performed technique was poor	41	60	36	29	NS
3	Examined patient over gown	30	33	30	35	NS
4	Approach lacks structure or order	26	11	38	21	NS
5	Rushed through examination; did not use available time	14	14	15	6	NS
						
2B. Physical examination: cognitive problems						
1	Inadequate data gathering to rule out other possible diagnosis	69	91	62	71	NS
2	Employed examination maneuvers not indicated by the case	10	14	10	6	NS
			Prevalence by case			
			
3. Professionalism		Prevalence (%)	Case 1 (%)	Case 2 (%)	Case 3(%)	Probability

1	Student did not introduce self to patient	33	29	30	41	NS
2	Treated SP as diagnostic problem rather than person with feelings and concerns	4	7	0	5	NS
3	Student did not wash hands	4	0	10	5	NS
4	Poor verbal and non-verbal communication (emotional distance)	2	7	0	0	NS
5	Student dressed inappropriately or casually	0	0	0	0	NS
						
4.	Overall assessment: failure of clinical encounter	74	100	64	76	.040
Sample size: number of clinical encounters		82	11	18	16	

In terms of the physical examination, missing required elements (69%) and inadequate data gathering to rule out other diagnoses (69%) were the most common problems identified. In contrast to the medical interview, the most common problems associated with the physical examination were problems related to execution, such as poor technique (41%), examining the patient over the gown (30%), and lack of structure or order (26%). In terms of Professionalism, relatively few problems were identified; however in one-third of the encounters, the students failed to adequately introduce themselves to their patients.

### Testing different scoring models

One of the goals of this study was to determine if faculty ratings could reproduce the final examination results based on SP ratings. Two approaches were considered in scoring the faculty ratings. For Model A, the student is given the benefit of the doubt and one rating of satisfactory is sufficient. Under this scheme, student performance was not considered problematic if one or both faculty members rated the performance as satisfactory; if neither rater gave a rating of satisfactory then performance was judged unsatisfactory. For Model B, both raters had to indicate satisfactory performance for a student to have demonstrated competency. A student's performance was problematic if one or both of the raters indicated it unsatisfactory. For both models, a rating of marginal was considered as unsatisfactory, that is, a marginal student was considered as not demonstrating minimal competency.

Under Model A, 74% of the encounters selected as potential failures based on SP ratings were judged as failures – marginal or unsatisfactory – by our faculty raters. Using Model B, 93% of the students considered failing the examination based on SP ratings were also considered not achieving competency based on the faculty ratings.

### Variability of performance problems

The variability of performance problems is shown in Table 2, which compares the frequency of problems identified across three different cases used in the clinical encounters that generated the largest number of student failures. These cases represented an undifferentiated complaint (Case 1), post-operative pain (Case 2), and headache (Case 3). When the frequency of problems was compared across cases and tested using a Chi-square statistic, 7 out of the 14 technical and cognitive problems associated with the medical interview were found to vary by case. In contrast, none of the problems associated with the physical examination or professionalism were found to vary across the three cases, suggesting more uniform skill performance across cases.

## Discussion

The purpose of this article is to describe the development of a worksheet that faculty could use to diagnose the performance problems of medical students, determine the prevalence of performance problems common to third-year medical students, and compare pass/fail decisions based on SP versus faculty ratings. The clinical encounters analyzed in this study were derived from students who did not meet passing standards based on standardized patients’ ratings. As the encounters were chosen on the basis of poor performance, it was expected that they would yield a high frequency of performance problems. These are students typically identified for remediation and require additional instruction and practice. The use of two independent faculty ratings was considered as two independent expert reviews; the paired ratings were able to reproduce the examination outcomes based on SP ratings to a high degree of accuracy although the rating tasks of the two groups were very different. The SP ratings were based on very specific case-related features of student performance, whereas the faculty ratings were derived from more global performance factors.

Our findings highlight the relative likelihood of various performance problems, and that these common problems are representative of all three of the major performance domains under consideration: medical interview, physical examination, and professionalism. Furthermore, the comparison of skills across cases suggests that some domains of clinical performance are more generalizable than others: performance problems associated with the medical interview were more case-specific than problems related to the physical examination or professionalism. Information about performance problems common to large groups of students or specific clinical encounters can provide an important source of curricular feedback for enhancing curriculum content, remediation planning for students not demonstrating competency and faculty development related to instruction and feedback. Our medical school is now instituting a broad-based faculty development initiative to alert community preceptors to these common performance problems and to suggest methods for providing corrective feedback to students.

The positive outcomes outweighed the faculty-intensive nature of this process. Recruiting clerkship faculty to participate as raters provided the same individuals responsible for implementation of the curriculum with the opportunity to observe first hand a significant sample of student performance in standardized encounters. These faculty members represent an important part of closing the loop on data collection. Their participation in this process provided them with direct feedback about strengths and weaknesses of student performance and more importantly, included insights into how these findings can be used to strengthen our educational program.

Since the development and initial testing of this worksheet, it has been used by faculty observers for subsequent clinical competency examinations to characterize the strengths and weaknesses of student performance. In addition, it has been used for new case development. As new cases are pilot tested in the Year 3 OSCE and the *a priori* performance standards are applied to the SP checklist ratings, the clinical encounters of all students who would fail the examination are reviewed by pairs of faculty using this worksheet to validate the decisions based on the SP ratings. This approach provides reassurance to the faculty that students identified as failing the clinical competency examination based on SP ratings demonstrated significant performance problems. Furthermore, we are hopeful that the experience of using this performance problem worksheet will provide faculty with an additional tool for diagnosing individual student performance problems and developing individualized educational plans to meet student-learning needs.

This study had several limitations. The students in this study were from a single institution and represent those students most likely to demonstrate performance problems. In addition, not all of the problematic clinical encounters were reviewed by two faculty members and thus were excluded from the study. While there is no reason to believe that the unrated cases were different in any systematic way, the factors influencing the resultant sampling strategy are unknown.

The development of this worksheet represents a step forward in a long-term goal of moving beyond the highly specific communication, history-taking, and physical examination skills checklists that are based on specific clinical cases. This worksheet allows faculty to characterize a learner's performance problems, which is a necessary first step for the learner to be able to address the problem (10). It provides faculty with a more standardized frame-of-reference for documenting and discussing performance problems than the frequent ‘I know it when I see it’ approach. However, requiring faculty to make decisions about satisfactory and unsatisfactory performance reinforces that this is an expert judgment task and that these types of decisions are expected of faculty. This worksheet places less emphasis on specific features of the case and focuses more on problems of medical students’ approach to data collection and problem solving as a means of giving students meaningful feedback while maintaining the security of SP cases. For individual students, this strategy provides information that can be used as corrective feedback to guide practice and strengthen performance. For faculty, this standardized approach for gathering evaluative information can be used to enhance curricular instruction and assessment as well as inform remediation efforts. This worksheet continues to be used by faculty for reviewing substandard OSCE performance as a framework for structuring formative feedback. It is also used for the reviewing the results of newly developed cases to validate that students identified as failing based on SP ratings indeed have failed to meet the performance standards of the cases. A number of possible refinements to the worksheet are being considered. This includes rewording some of the items to enhance clarity, changing the Section III heading to ‘Professionalism’ to better reflect the content, and adding prompts to Section IV where faculty can list three areas where the student performed well.

As a next step, it would be instructive to investigate the extent to which high-performing students as determined by the SP checklists demonstrate performance problems from this ratings worksheet. As part of the faculty training experience described in this article, each of the two clinical encounters that received high SP checklist scores also had three or four performance problems, again supporting the differences between types of information derived from skill-specific checklists completed by standardized patients as compared to more global ratings by faculty. Information about the performance problems sampled from students at all levels could further clarify appropriate expectations for learners, and provide curricular feedback about the effectiveness of instruction and assessment, as well as highlight the appropriate use of ratings based on specific versus more global items for learners of various levels.

## Appendix

Rating form

Student performance worksheet

I. MEDICAL INTERVIEW

II. PHYSICAL EXAMINATION

III. TEAMWORK AND PROFESSIONALISM

IV. FINAL ASSESSMENT OF OVERALL STUDENT PERFORMANCE ON CASE

Issues to address in remediation:

1.

2.

3.

[_] Check here if the technical quality of the recording was problematic: [_] Audio [_] Video
